# Clinical Effects of Topical Tacrolimus on Fox-Fordyce Disease

**DOI:** 10.1155/2015/205418

**Published:** 2015-06-15

**Authors:** Hilal Kaya Erdoğan, Işıl Bulur, Zeliha Kaya

**Affiliations:** ^1^Department of Dermatology, Eskisehir Osmangazi University, 26480 Eskisehir, Turkey; ^2^Department of Pathology, Kırsehir Ahi Evran University, 40200 Kirsehir, Turkey

## Abstract

Fox-Fordyce Disease (FFD) is a rare, chronic, pruritic, inflammatory disorder of apocrine glands. It is characterized by dome-shaped, firm, discrete, skin-colored, and monomorphic perifollicular papules. The most common sites of involvement are axillae and anogenital and periareolar regions which are rich in apocrine sweat glands. Treatment is difficult. Topical, intralesional steroids, topical tretinoin, adapalene, clindamycin, benzoyl peroxide, oral contraceptives, isotretinoin, phototherapy, electrocauterisation, excision-liposuction and curettage, and fractional carbon dioxide laser are among the treatment options. In the literature, there are articles reporting beneficial effects of pimecrolimus in FFD. Nevertheless, there have not been any reports about the use of tacrolimus in FFD. We report two patients diagnosed with FFD by clinical and histopathologic examination and discussed therapeutic effects of topical tacrolimus on FFD in the light of literature.

## 1. Introduction

Fox-Fordyce Disease (FFD) or “apocrine miliaria” is a chronic, pruritic, rare, inflammatory disorder of apocrine glands. It is observed primarily in women between the ages 15 and 35 and usually remits after menopause [1–3]. There are few reports of prepubescent patients in the literature [4].

Clinically it is characterized by dome-shaped, firm, discrete, skin-colored, and monomorphic perifollicular papules. Most common sites of involvement are axillae, anogenital, and periareolar regions which are rich in apocrine sweat glands. Less common locations include the medial thighs and periumbilical and sternal regions. The affected areas show reduction of sweating and hairs. The chief complaint generally is severe pruritus. Exercise, heat, and emotional stress can aggravate pruritus [1, 2].

Herein we report two patients diagnosed with FFD and discuss therapeutic effects of topical tacrolimus in the light of literature.

## 2. Report of Cases

### 2.1. Case 1

**Figure 1:**
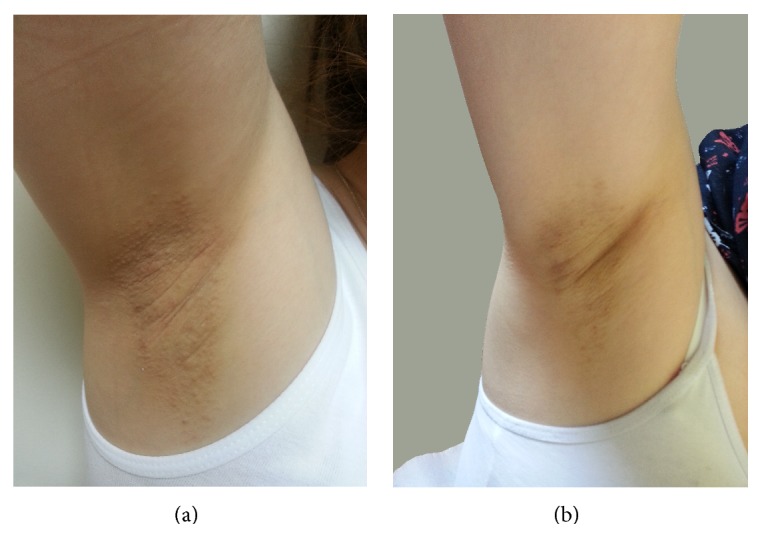
(a) Before treatment and (b) improvement of lesions after 3 months of topical tacrolimus.

A 23-year-old woman presented with intensely pruritic lesions on her axillae for 3 years. She had been previously unsuccessfully treated with topical steroids, antifungals, and antibiotics. Her medical and family history was unremarkable. Dermatological examination revealed multiple, monomorphic, perifollicular, firm, skin-colored, and hyperpigmented papules confined to the bilateral axillary areas (Figure 1(a)). The remainder of her physical examination results was unremarkable. Histology from an axillary skin biopsy revealed hyperkeratosis and keratotic plug in follicular infundibulum, spongiosis, lymphocyte exocytosis, and perivascular and periadnexal lymphocytic infiltration. The diagnosis of FFD was made by clinical and histopathological findings. She was prescribed topical tacrolimus ointment (0,1%) twice daily for 3 months. After 3 months she had marked improvement of her lesions and pruritus (Figure 1(b)). There were no side effects of the treatment.

### 2.2. Case 2

**Figure 2:**
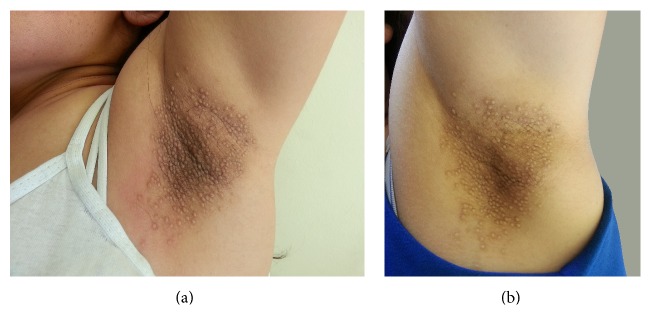
(a) Before treatment and (b) no change after 3 months of topical tacrolimus.

**Figure 3:**
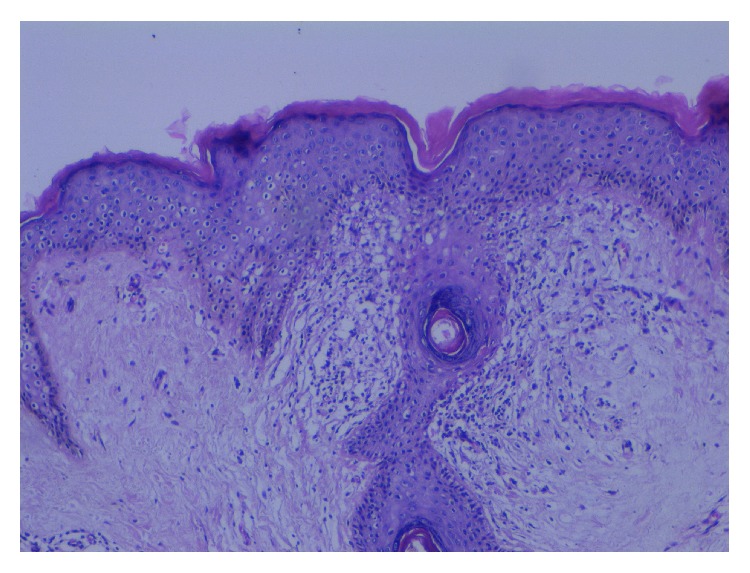
Hyperkeratosis, a keratotic plug in the follicular infundibulum, spongiosis, lymphocyte exocytosis, and perivascular and periadnexal lymphocytic infiltration.

A 32-year-old woman presented with papular lesions on her axillae for 10 years. Although the disease in this patient was subjectively asymptomatic, lesions were cosmetically disfiguring. She had been previously unsuccessfully treated with topical steroids. Dermatological examination revealed multiple, monomorphic, perifollicular, firm, skin-colored, and hyperpigmented perifollicular papules confined to the bilateral axillary areas. Also thinning of axillary hair was noted (Figure 2(a)). The remainder of her physical examination results were unremarkable. Histologic examination of a 4 mm punch biopsy specimen taken from one of the papules revealed marked hyperkeratosis and keratotic plug in follicular infundibulum, spongiosis, lymphocyte exocytosis, and perivascular and periadnexal lymphocytic infiltration (Figure 3). The diagnosis of FFD was made by clinical and histopathological findings. She was prescribed topical tacrolimus ointment (0,1%) twice daily for 3 months. After 3 months, there was no change in lesions, and treatment was stopped (Figure 2(b)).

## 3. Discussion

FFD, first described by George Henry Fox and John Addison Fordyce in 1902, is a rare, pruritic, inflammatory disease of apocrine glands [2].

Etiology is not completely known. However, female predominance, start of symptoms with the onset of puberty, flare up in perimenstruel period, regress in pregnancy, post-menopausal period and by using oral contraceptives indicate hormonal factors. On the other hand, prepubertal FFD cases, lack of hormonal abnormalities, monozygotic twin, and familial case reports suggest that genetic and emotional factors may play role in etiology [2, 4, 5]. Besides, in literature, reported FFD cases after axillary hair removal suggest that physical factors also may play role [6].

Although the chronology of events is not proven in pathogenesis, the mechanism defined by Shelley and Levy is widely recognized. First event is obstruction of apocrine canal's distal part with keratin plug. It is considered that the keratin plug occurred by dysmaturation of keratinocytes. Canal is ruptured in epidermis because of apocrine sweat retention. This is followed by perifollicular and periadnexal infiltration [3, 6, 7].

Early histological finding is the keratin plug that blocks apocrine ductus in follicular infundibulum. Epidermal spongiosis and vesiculation occur. Perifollicular and periadnexal infiltration which consists of lymphocytes, few histiocytes, and eosinophils is accompanying finding. Mataix et al. noted that xanthomatosis due to phagocytosis of fat-rich apocrine material by macrophages was also an important histopathologic finding [3, 7].

Topical and intralesional steroids are first-line treatment in FFD, but their use is limited due to risk of cutaneous atrophy and striae. Topical tretinoin, adapalene, clindamycin, benzoyl peroxide, and pimecrolimus are used in the treatment. Oral contraceptives and oral isotretinoin treatment are also tried in the treatment. Phototherapy, electrocauterisation, excision-liposuction and curettage, and fractional carbon dioxide laser are also among the treatment options [8–11].

Tacrolimus is a calcineurin inhibitor and is apparently more effective than pimecrolimus in atopic dermatitis. Although there are articles reporting beneficial effects of pimecrolimus in FFD, there have not been any reports about the use of tacrolimus in FFD. We used tacrolimus pomade (0,1%) on our patients twice a day due to its strong anti-inflammatory effect and little side effect profile. While the first patient was successfully treated with tacrolimus, we did not observe any improvement in the second one. We think that the first patient responded well because she had more inflammatory disease with newer lesions and intense pruritus. On the other hand, the failure of the treatment in second patient can be explained by long duration of disease and marked keratinization. The difference in treatment response between two patients reminds us of the question that either keratin plug formation or inflammation is the primary pathogenetic event in pathogenesis [10, 11].

Consequently, topical calcineurin inhibitors should be noted as an option in the treatment of FFD patients with intense pruritus and short disease duration rather than patients with more chronic course and prominent keratinization.

## References

[1] Schaller M., Plewig G. (2008). Structure and function of eccrine, apocrine, apoeccrine and sebaceous glands. *Dermatology*.

[2] Yost J., Robinson M., Meehan S. A. (2012). Fox-Fordyce disease. *Dermatology Online Journal*.

[3] Arca E., Köse O., Taştan H. B. (2003). A case of Fox-Fordyce. *Turkiye Klinikleri Journal of Medical Sciences*.

[4] Demirci G. T., Yaşar Ş., Mansur A. T., Aydingöz I. E., Sever S. (2006). Prepubertal Fox-Fordyce disease: a case report. *Turkiye Klinikleri Journal of Medical Sciences*.

[5] Erkek E., Koçak M., Atasoy P. (2002). Fox-Fordyce disease. *Turkderm*.

[6] Tetzlaff M. T., Evans K., DeHoratius D. M., Weiss R., Cotsarelis G., Elenitsas R. (2011). Fox-Fordyce disease following axillary laser hair removal. *Archives of Dermatology*.

[7] Mataix J., Silvestre J. F., Niveiro M., Lucas A., Pérez-Crespo M. (2008). Perifollicular xanthomatosis as a key histological finding in Fox-Fordyce disease. *Actas Dermo-Sifiliograficas*.

[8] Kassuga L. E. D. B. P., Medrado M. M., Chevrand N. S., Salles S. D. A. N., Vilar E. G. (2012). Fox-Fordyce disease: response to adapalene 0.1%. *Anais Brasileiros de Dermatologia*.

[9] Chae K. M., Marschall M. A., Marschall S. F. (2002). Axillary Fox-Fordyce disease treated with liposuction-assisted curettage. *Archives of Dermatology*.

[10] Milcic D., Nikolic M. (2012). Clinical effects of topical pimecrolimus in a patient with Fox-Fordyce disease. *Australasian Journal of Dermatology*.

[11] Pock L., Švrčková M., Macháčková R., Hercogová J. (2006). Pimecrolimus is effective in Fox-Fordyce disease. *International Journal of Dermatology*.

